# Host Specific Diversity in *Lactobacillus johnsonii* as Evidenced by a Major Chromosomal Inversion and Phage Resistance Mechanisms

**DOI:** 10.1371/journal.pone.0018740

**Published:** 2011-04-20

**Authors:** Caitriona M. Guinane, Robert M. Kent, Sarah Norberg, Colin Hill, Gerald F. Fitzgerald, Catherine Stanton, R. Paul Ross

**Affiliations:** 1 Food Biosciences Department, Teagasc Food Research Centre, Moorepark, Cork, Ireland; 2 Department of Microbiology, University College, Cork, Ireland; 3 Alimentary Pharmabiotic Centre, University College, Cork, Ireland; Swiss Tropical and Public Health Institute, Switzerland

## Abstract

Genetic diversity and genomic rearrangements are a driving force in bacterial evolution and niche adaptation. We sequenced and annotated the genome of *Lactobacillus johnsonii* DPC6026, a strain isolated from the porcine intestinal tract. Although the genome of DPC6026 is similar in size (1.97mbp) and GC content (34.8%) to the sequenced human isolate *L. johnsonii* NCC 533, a large symmetrical inversion of approximately 750 kb differentiated the two strains. Comparative analysis among 12 other strains of *L. johnsonii* including 8 porcine, 3 human and 1 poultry isolate indicated that the genome architecture found in DPC6026 is more common within the species than that of NCC 533. Furthermore a number of unique features were annotated in DPC6026, some of which are likely to have been acquired by horizontal gene transfer (HGT) and contribute to protection against phage infection. A putative type III restriction-modification system was identified, as were novel Clustered Regularly Interspaced Short Palindromic Repeats (CRISPR) elements. Interestingly, these particular elements are not widely distributed among *L. johnsonii* strains. Taken together these data suggest intra-species genomic rearrangements and significant genetic diversity within the *L. johnsonii* species and indicate towards a host-specific divergence of *L. johnsonii* strains with respect to genome inversion and phage exposure.

## Introduction

The Gastro-intestinal (GI) tract is colonized by a vast and diverse community of microbes. Lactobacilli represent an important part of the natural gut microbiome of both humans and animals and have been extensively studied for their health promoting properties. *Lactobacillus johnsonii* is a member of the closely related “acidophilus complex” of lactobacilli and an autochthonous species of the gastro-intestinal tract. *L. johnsonii* strains are of interest due to the number of probiotic characteristics associated with this species, including immunomodulation, [1], [2], [3], [4] attachment to epithelial cells [5], [6] and pathogen exclusion [7], [8], [9].

For organisms commonly found in GI tract such *Lactobacillus acidophilus*, *Lactobacillus gasseri* and *L. johnsonii* there are a number of genome sequences available which have identified genetic traits that most likely function in gastric survival and promote interactions with the intestinal mucosa [9], [10], [11], [12]. It has been proposed that GI-associated strains have adapted to their niche with a specialized set of metabolic and surface-related proteins [13]. In the *L. johnsonii* NCC 533 genome for example, large cell surface proteins were identified thought to be involved glycoprotein adhesion and persistence in the intestinal tract [12], [14]. A common trait documented also for this group of organisms is a general lack of genes encoding biosynthetic pathways for amino acids, purine nucleotides and cofactors which may be reflective of their “symbiont” nature and an abundance of ABC transporters, peptidases and phosphotransferases [10], [11], [12].

Genomic heterogeneity within a bacterial species can be driven by the selective pressure of different environmental niches and can result from recombination events and the presence of mobile genetic elements (MGE), such as bacteriophage and IS elements. Genetic diversity and horizontal gene transfer (HGT) among closely related gut lactobacilli has been observed [11], [12], [15], [16]. Within the ‘acidophilus complex’ previous polyphasic analysis and comparative genomic analysis has indicated significant inter and intra-species diversity among MGE and at the region around the terminus of replication [15]. The possibility of genomic rearrangements at this region within *L. johnsonii* strains has also been previously suggested [17] (Contribution by Pridmore D; [18]).

Here we present the whole genome sequence of the porcine *L. johnsonii* isolate DPC6026 (previously named *L. acidophilus* DPC6026; [19]) and explore the genetic content, the potential genomic rearrangements and diversity within the *L. johnsonii* species. This study also presents a number of MGE novel to the *L. johnsonii* species and previously unidentified phage resistance mechanisms.

## Materials and Methods

### Bacterial strains, growth conditions

*L. johnsonii* DPC6026 was originally isolated from a porcine small intestine [19]. This strain was previously identified as *L. acidophilus* DPC6026 however more refined 16S sequencing demonstrated that it belongs to the *L. johnsonii* species rather than the closely related *L. acidophilus*. All isolates used in this study are outlined in Table 1. Cultures isolated from faecal samples were as previously described [20] and screened on Lactobacilli selective agar (LBS). *L. johnsonii* strains were cultured anaerobically in MRS (Difco) media at 37°C.

**Table 1:** Strains used in this study.

Strain	Species	Source	Reference
aDPC6026	*L. johnsonii*	Porcine	[19]
DPC6092	*L. johnsonii*	Porcine	[19]
DPC6214	*L. johnsonii*	Porcine	[19]
DPC6560	*L. johnsonii*	Porcine	This study
DPC6561	*L. johnsonii*	Porcine	This study
DPC6562	*L. johnsonii*	Porcine	This study
DPC6563	*L. johnsonii*	Porcine	This study
DPC6564	*L. johnsonii*	Porcine	This study
DPC6565	*L. johnsonii*	Porcine	This study
NCC533	*L. johnsonii*	Human	[12], [60]
DSM10533	*L. johnsonii*	Human/Type strain	bDSM
ATCC120883	*L. johnsonii*	Human/Type strain	cATCC
LMG9433	*L. acidophilus*	Type strain	dLMG
ATCC4356	*L. acidophilus*	Human/Type strain	cATCC
DPC6489	*L. gasseri*	Human	[61]
LMG9203	*L. gasseri*	Type strain	dLMG

a DPC collection; Dairy Product Collection, Moorepark Food Research Centre, Fermoy, Co. Cork.

b DSM; DSMZ, Deutsche Sammlung von Mikroorganismen und Zellkulturen.

c ATCC; American Type Culture Collection

d LMG; BCCM/LMG Bacteria collection.

### Speciation of isolates

DNA was extracted from 10 ml overnight cultures using the procedure previously described [21]. The 16S rDNA were amplified from gDNA from each strain using species specific primers for *L. johnsonii, L. gasseri* and *L*. *acidophilus* as previously described [22]. Chosen isolates were confirmed by amplification using 16S Eubacterial primers [23] and the 16S region was sequenced by conventional Sanger sequencing. The species was determined by nucleotide alignments (>98%) with deposited species in the NCBI database. Strains of the same species were confirmed to be different isolates by Pulsed-Field-Gel-Electrophoresis using the *apaI* enzyme (not shown).

### Phylogenetic analysis

Reconstruction of evolutionary relationships were carried out using the MEGA 4 package [24]. 16S rRNA sequence data was obtained from GenBank (*L. johnsonii* AE017198, *L. gasseri* CP000413, *L. acidophilus* CP000033, *Lactobacillus sakei* CR936503, *Lactobacillus reuteri* CP000705, *Lactobacillus fermentum* AP008937, *Lactobacillus brevis* HQ622718, *Lactobacillus plantarum* CP002222, *Lactobacillus salivarius* CP000233 and *Lactobacillus casei* FM177140) and was used to construct a consensus neighbour joining tree from 500 bootstrapping replicates.

### Genome sequencing, assembly and comparative genomic analysis

Massively parallel 454 pyrosequencing with paired end tags of DPC6026 to a coverage of 23X was performed by 454 Beckmann Coulter Genomics (www.beckmancoulter.com) on a FLX sequencer followed by initial assembly in to 83 contigs using the Newbler program (roche-applied-science.com). Order and orientation of assembled contigs and predicted scaffolds was determined using the published genome sequences of *L. johnsonii* NCC533 [12] and *L. johnsonii* FI9785 [9]. Primers were designed at gap edges using primer3 [25] for PCR amplification of gap regions using Platinum Hi-fidelity PCR Supermix (Invitrogen) or Kod DNA Polymerase (Novagen). Reactions were performed in a Biometra TGradient followed by directed sequencing of PCR products by primer walking, and whole genome assembly was performed using the PHRED-PHRAP-CONSED package [26], [27]. Raw assembly reads were visualised and verified using the programme Hawkeye (Amos) [28]. Unmapped contigs were mapped using combinatorial PCR followed by primer walking. Frameshifts and ribosomal operons were annotated but not verified by conventional Sanger sequencing.

Coding regions were predicted using Glimmer 2 [29] and annotation was performed using GAMOLA [30]. Complementary annotation data were provided by the SEED [31] and the RAST annotation server [32]. Data was manually curated (Oct 2010) using Artemis software V11 [33] where additional programmes were used including, PROSITE (www.expasy.ch) RBS finder [29] and GATU [34]. Comparative genomics was performed using the Artemis comparison tool [35] and MAUVE software [36]. Circular maps were created using DNA plotter [37].

### Detection of novel features of DPC6026

Primers specific to the regions of genomic rearrangements were designed based on the genome sequences of DPC6026 and NCC533 (Table S1). Primers specific to 4 regions of the integrated prophage and the site of prophage integration were designed based on DPC6026. The primers used to detect the CRISPR elements and restriction modification systems were designed based on DPC6026 with at least 2 different specific combination of primers used (Table S1). PCRs were performed on all strains (Table 1) to confirm genomic structure and elements using either Platinum Hi-fidelity PCR Supermix (Invitrogen) or Biotaq (Bioline).

### Phage Induction

The induction of the prophage Фlj6026 was attempted by heat where the culture containing the phage was subjected to a thermal stress of 42°C for 1 hour or following the addition of mitomycin C (2–6 µg/ml) (Sigma Chemical Co., St. Louis, MO). *L. johnsonii* was grown overnight in MRS broth at 37°C anaerobically. Fresh broth was inoculated with a 1% inoculum of the overnight strain and grown to OD600_nm_ 0.1–0.3. The culture was centrifuged and the supernatant was filtered through a 0.45 µm filter. The filtered supernatant was spotted on an overlay of a range of indicator strains and prophage release was determined by observing zones of lysis following incubation at 37°C for 24 h (Table 1).

### Public data sources

The genome sequence of *L. johnsonii* DPC6026 is available from GenBank/EMBL under the accession number CP002464.

## Results

### General features of the genome of *L. johnsonii* DPC6026

**Figure 1:**
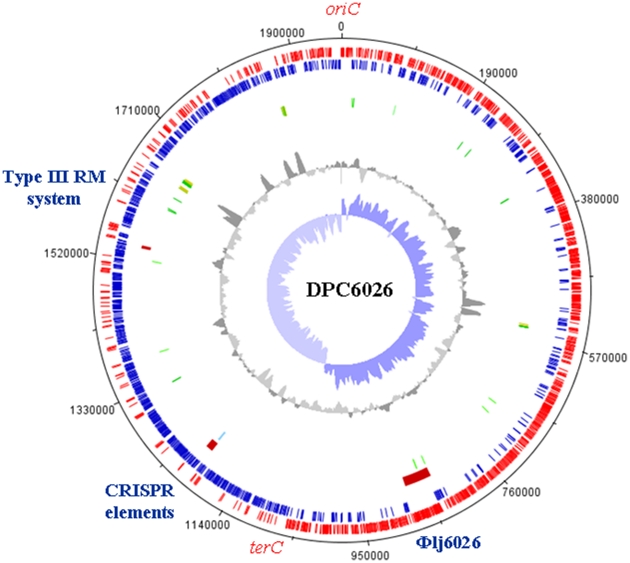
Genome Atlas of *L. johnsonii* DPC6026. The tracks from the outside represent 1. Forward CDS, 2. Reverse CDS, 3. Misc. features/MGE, 4. tRNA, rRNA 5. % GC plot 6. GC skew.

The DPC6026 genome consists of a singular circular chromosome of 1.97 mbp with an average G+C content of 34.8% and does not harbour any plasmids (Figure 1). Overall, the genome of DPC6026 was highly similar to the previously sequenced members of the species *L. johnsonii* in size, G+C content and gene synteny [9], [12]. Total GC-skew analysis and the ORF orientation drift identified the *oriC* proximal to *dnaA* and the *terC* at ∼1.05 mb (Figure 1). *In silico* analysis predicted 1795 protein coding genes.

**Figure 2:**
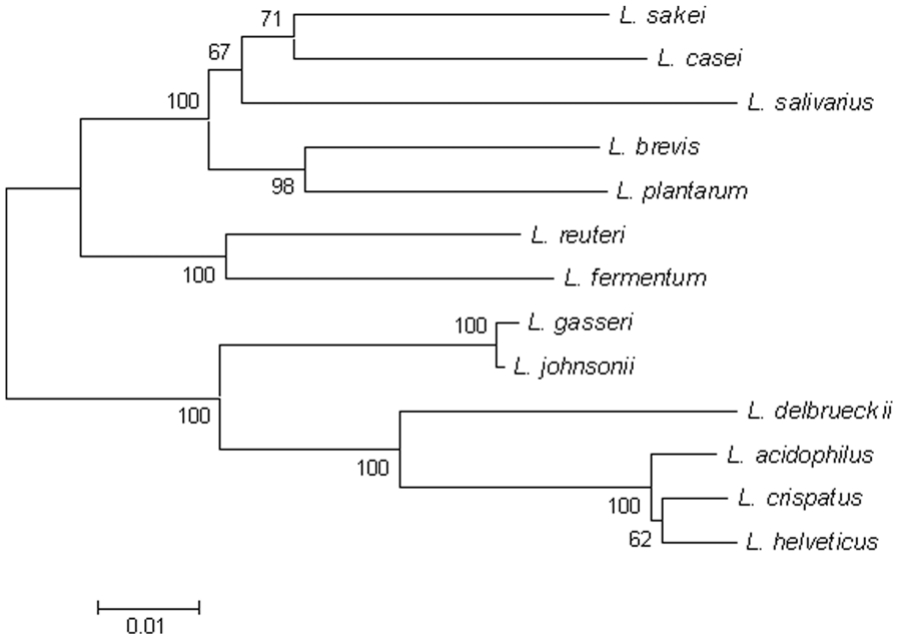
Phylogenetic tree based on the 16S rRNA gene sequences of *Lactobacillus* species.

Phylogenetic analysis based on the 16S rRNA gene sequences of *L. johnsonii* and other Lactobacilli revealed, in accordance with previous work [38], that *L. johnsonii* is closely related to other *L. acidophilus* complex members (Figure 2). It is most related however to the gut bacterium *L. gasseri* as they occupy the same branch on the phylogenetic tree (Figure 2).

### Genetic homogeneity of the core genome of *L. johnsonii* sequenced isolates

Among the genes encoded in DPC6026, 150 genes (∼9%) were not found in the human isolate NCC 533 [12], 84 genes (5%) were novel to the *L. johnsonii* species and just 18 (1%) genes were not previously identified in the genus *Lactobacillus*. These results are in accordance with previous work by Berger et al., (2007) which indicated a conservation of genes between *L. johnsonii* isolates to be between 83–92% with 5% strain specific genes [15]. Genes novel to DPC6026 largely represented mobile DNA including genes encoding proteins with homology to phage related proteins, transposase and insertion elements.

The metabolic capabilities and biosynthetic pathways of DPC6026 are in accordance with the reliance of *L. johnsonii* on the surrounding environment for nutrients [12]. DPC6026 has a high number of PTS systems and ABC transporters enabling utilization of sugars available in the GI tract, similar to the closely related genomes of the ‘acidophilus complex’ [10], [11], [12]. There were also 20 proteins with homology to peptidases annotated in the DPC6026 genome, including eight aminopeptidases, six dipeptidases and three endopeptidases. This is in agreement with the dependency of the *L. johnsonii* on exogenous amino acids for growth. The extracellular cell wall bound proteinase (LJ1840) that was annotated in NCC533 however was not found in the porcine strain. PCR analysis indicated that this was not present in any of the porcine isolates tested (not shown). This was surprising as the *L. johnsonii* DPC6026 strain was previously indicated to have proteolytic ability [19] and it was reported that DPC6026 generates antimicrobial peptides from casein in milk-based fermentations [19]. However, our phenotypic analysis supports the genomic prediction that this strain alone cannot hydrolyse milk efficiently and further analysis to the possibility of indigenous microbiota from the fermentation substrates contributing to proteolysis and the liberation the antimicrobial peptides is ongoing.

The abundance of transport and regulatory proteins is also reflected in the genomes of *L. johnsonii* NCC533 [12] and FI9785 [9], however, there were differences in the genetic content of these proteins within each of the three genomes. These differences may be due to a differing GI environment among the disparate host species. Of note also is the differing complement of adhesion and cell surface proteins present in DPC6026 and in NCC533. Pridmore *et al*., (2004) identified cell surface components (LJ0382, LJ0391, LJ1128, LJ1711, LJ1839) in the human isolate thought to be unique to NCC 533 and predicted to be secreted and attached to the cell surface. These proteins were all either absent or appeared to be fragmented (LJP0353, LJP0366, LJP0707 and LJP1463) in the porcine isolate. This could further indicate the importance of these proteins in colonisation of a human host.

### Genome Architecture and Synteny

**Figure 3:**
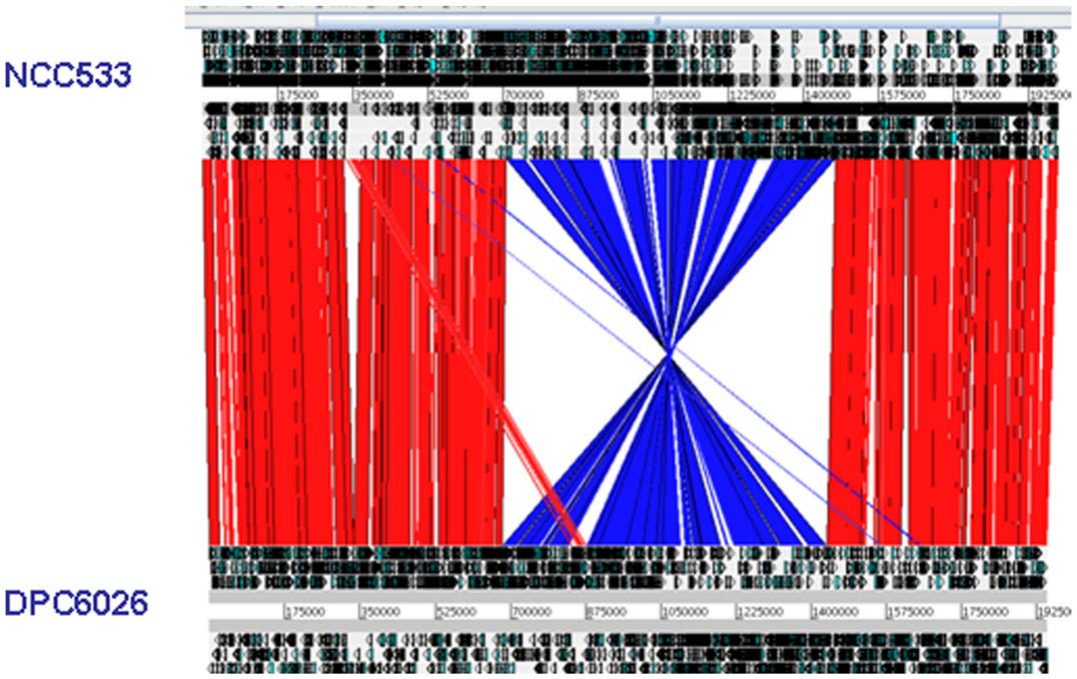
Pairwise comparison of the chromosomes *L. johnsonii* DPC6026 and NCC 533 using ACT (a). The sequences have been aligned from the predicted replication origins (*oriC*). The colored bars separating each genome (red and blue) represent similarity matches identified by BlastN analysis, with a filter cutoff of 100. Red lines link matches in the same orientation; blue lines link matches in the reverse orientation.

Despite a relatively conserved gene synteny between the sequenced *L. johnsonii* isolates, there is a large (∼750 kb) symmetrical inversion across the replication axis between the human isolate NCC 533 and the porcine isolate DPC6026 (Figure 3). Whole genome alignments also indicate that the porcine isolate DPC6026 and poultry strain FI9785 share the same genomic arrangement (not shown).

**Figure 4:**
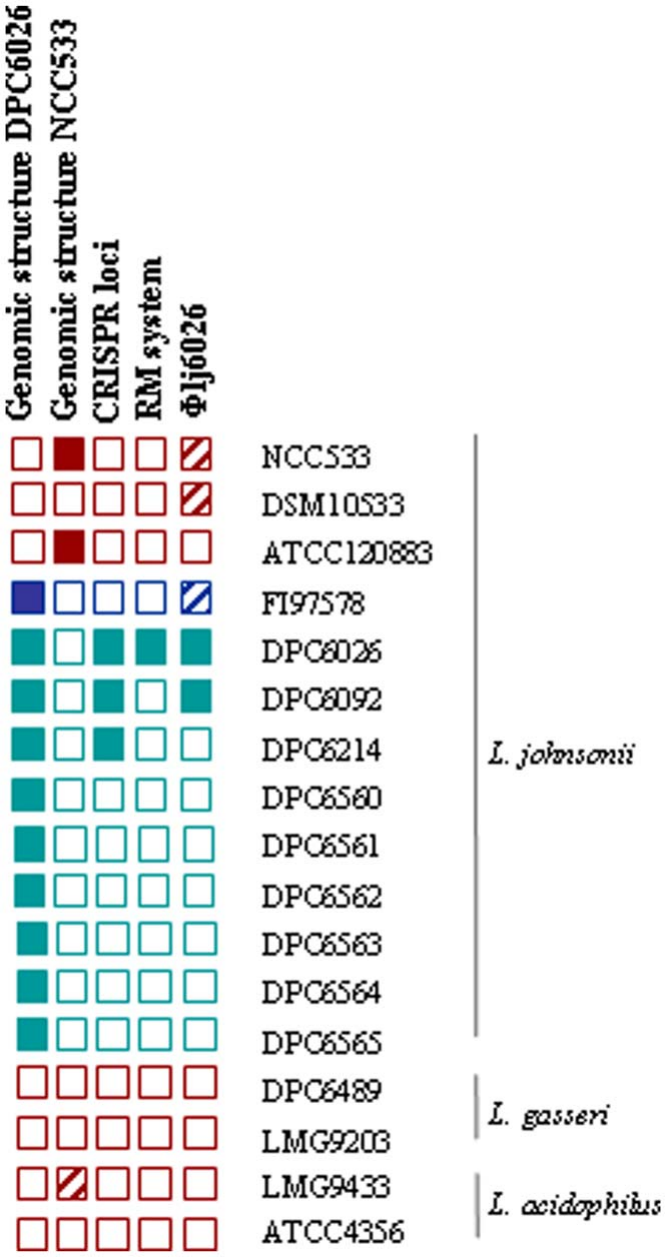
Comparative genomics of *Lactobacillus* strains. The distribution of the genomic inversion, the CRISPR loci, the type III restriction modification system and the integrated prophage among a panel of *L. johnsonii* isolates of human (red), poultry (blue) and porcine (green) origin, *L. gasseri* and *L. acidophilus* strains. A filled square indicates presence of the element, a hatched square indicates a partial element and an empty square indicates the element in absent.

Despite the large genomic inversion, the *ori* and *ter* regions do not appear to be disrupted based on the location of the inversion and on the GC-skew data. Indeed, while a slightly imbalanced replichore is evident, there is not a significant change in the replichore sizes of the two strains (Figure 1). The existence of the inversion also did not lead to a significant difference in the growth rate of the strains (not shown). The inversion between DPC6026 and NCC 533 was confirmed by site-specific PCR. Two primer pair sets were designed that overlap the left and right junction sites in DPC6026 and yield an amplicon in this strain but should not in NCC 533 if this region had undergone an inversion. When the 2 primer sets are used in the combination (F/F) and (R/R), a PCR product is generated in NCC 533 but not in DPC6026, thus confirming the differing genomic structures and an inversion event (Figure S1). The genomic structure of 8 further porcine isolates and 2 human isolates of *L. johnsonii* was investigated using these primer sets. Results indicate all the porcine isolates harboured the same genomic structure as DPC6026. One human isolate, a type strain ATCC12088, harboured the same genomic arrangement as NCC 533 (Figure 4). The second human/type strain tested did not give a PCR product for either structure.

At both the right and left junction sites in NCC 533 a 1,460 bp sequence of inverted repeats was identified including an insertion element ISLjo2 of the ISL3 family which may be responsible for the inversion event in NCC 533 or in an ancestral strain (Figure S1). It has been documented that recombination involving direct repeats can lead to genomic inversions [39] and has been suggested previously as a possibility for the NCC 533 strain [18]. Differing genomic structures are also apparent on alignments of *L. johnsonii* strains with the closely related *L. gasseri* (Figure 2) (4, 11) indicating rearrangements in this group of bacteria can occur frequently and ‘X-shaped’ inversions across the replication terminus between species of the acidophilus group have been documented [15].

Based on the comparative genomic PCR assays it is likely that the structure of DPC6026 is the more commonly found genomic structure of *L. johnsonii*. The repeat region and IS element present in NCC 533 was not present at this location in DPC6026 but was however at 4 other locations within the porcine genome. Given that this is a common element in *L. johnsonii* genomes it may be an indication of significant genome plasticity within the species.

### Novel Mobile Genetic Elements of *L. johnsonii*

**Figure 5:**
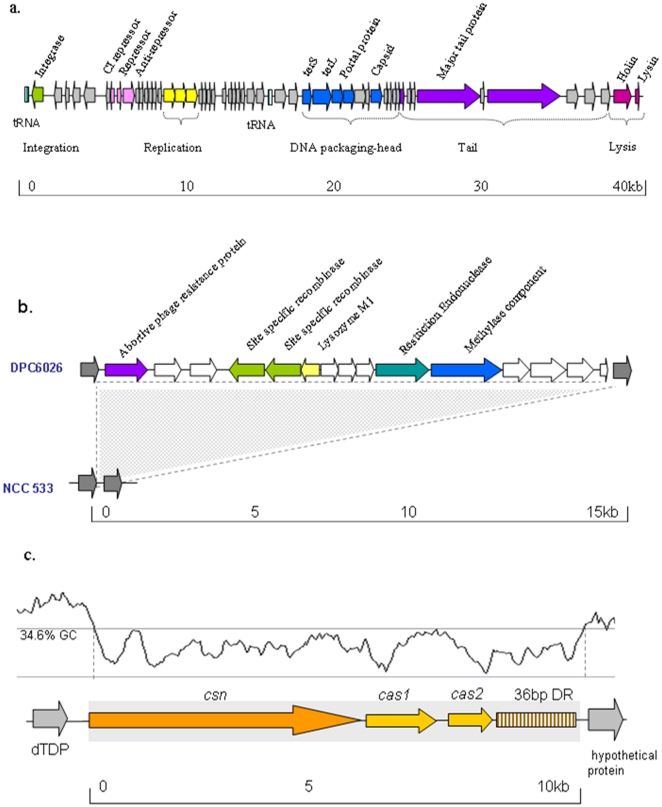
Graphic representation of the phage Фlj6026 (a), the restriction modification (RM) system (b) and the CRISPR loci (c) in the genome of DPC6026. Genes within a mobile element that are annotated to have a similar function are coloured the same. The RM system of DPC6026 (LJP1432-LJP1446) is absent from NCC 533 where hypothetical proteins LJ1697 and LJ1698 are shown by grey arrows. The CRISPR (*csn*) gene is represented by dark orange arrow and CRISPR-asocciated genes (*cas1*, *cas2*) are represented by light orange arrows. Repeat/spacer region (36 DR; Direct Repeats) are represented by brown lines. The entire CRISPR associated region is represented by a filled grey rectangle corresponding to the lowered GC content as predicted by Artemis (33).

Acquisition of genes by HGT is considered a major driving force in bacterial evolution and can impact on genomic structure and stability. Laterally acquired DNA provides a readily available pool of genes for developing physiological properties that are helpful in a particular niche. A number of previously unidentified MGEs were identified in the DPC6026 genome (Figure 5a, 5b and 5c).

#### (i) Integrated prophage

Prophages of *L. johnsonii* have been previously characterised [40], [41] and are indicated to have large role in the diversification within the species [17], [42]. Genomic analyses revealed the presence of one complete prophage sequence, Фlj6026 (LJP0764-LJP0819), which is integrated next to tRNA loci at ∼900 kbp within the DPC6026 genome. Of note this prophage is within the region that is inverted relative to the human isolate, however is integrated in the opposite orientation (Figure 3).

Фlj6026 is 43,608 bp in length and encodes 56 proteins comprising the typical phage regions of integration, replication, packaging, structural and lysis domains (Figure 5a). Фlj6026 phage shares an integration site with the NCC533 phage Фlj928 but most nucleotide identity with the NCC533 phage Фlj965 [40]. We attempted to induce Фlj6026 by mitomycin C and heat treatments using the closely related *L. johnsonii*, *L. acidophilus* and *L. gasseri* strains as indicator organisms. Release of the prophage was not detected by the methods used. The apparent non-functionality of Фlj6026 is in accordance with previous work that has indicated that the related prophages Фlj965 and Фlj928 are not inducible [40]. Distribution of Фlj6026 was investigated among *L. johnsonii* strains and strains of the closely related species *L. gasseri* and *L. acidophilus* (Figure 4). Of the isolates tested only the porcine *L. johnsonii* isolate DPC6092, in addition to DPC6026, appeared to harbour the full phage. Partial matches were obtained with the human type strain DSM10533. Based on *in silico* analysis, the poultry isolate, FI9785 was found to also have a similar but not identical phage within the genome (Figure 4).

#### (ii) IS Elements

IS elements are recognisable by DNA recombination machinery and can play a large role in chromosomal rearrangements. The annotation of DPC6026 identified 51 gene features with similarity to either characterized or predicted transposases or to putatively truncated or degenerate transposase enzymes. The type of IS elements differed considerably among the *L. johnsonii* sequenced isolates. In DPC6026, insertion elements of the family IS1223 that had been identified in NCC 533 and FI9785 were found in addition to copies of IS605 of in *L. acidophilus* NCFM [10] and ISLhe1 of *L. helveticus* DPC4571 [43].

#### (iii) Restriction Modification System

Restriction Modification (RM) systems function to cleave foreign DNA and are the most common systems used to degrade incoming phage DNA. A novel restriction modification system was annotated on the genome of DPC6026. It is located at ∼1.57 mbp and consists of a restriction (LJP1436) and a methylase (LJP1437) component typical of the type III family of RM systems (Figure 5b). This type III system has not been previously identified in *L. johnsonii* and, although it does share amino acid identity with the restriction component of *L. gasseri* (90%) [11] and the modification component of *Lactobacillus fermentum* (55%) [44], the complete system does not have a close homolog in any sequenced LAB. The type III R/M system is located in a ∼15 kb region (LJP1432-LJP1446) that is absent from NCC 533 (Figure 5b). This region also contains a protein (LJP1446) with identity (30% amino acid) to abortive phage resistance proteins which suggest a combination of different phage defence mechanisms present. Comparative genomic analysis indicated that this element is not widely distributed as it was not found in any of the other strains tested in this study (Figure 4).

### Analysis of the CRISPR locus in DPC6026

Clustered regularly interspaced short palindromic repeats (CRISPR) represent a family of DNA repeats shown to provide acquired immunity against foreign genetic elements [45], [46]. A novel CRISPR-cas system of 6.1 kb was identified in the genome of the porcine isolate. This element is positioned at the centre of the region that is inverted relative to NCC 533. A slight alteration in GC content compared to the surrounding region suggests that this element was transferred by horizontal gene transfer (Figure 5c).

CRISPR systems have been identified in nine *Lactobacillus* genomes to date [47], including closely related members of the acidophilus complex, *L. acidophilus* [10] and *L. helveticus* [43]. Despite this, the content of the CRISPR loci (LJP1108-1110) in *L. johnsonii* was not identical when compared to elements in closely related organisms. Differences within the repeat region and in the CRISPR associated (Cas) proteins were also observed. The 36 bp repeat 5′ATCTAAACCTTATTGATCTAACAACCATCTAAAAC3′ is present 28 times with 27 unique spacer sequences. The three genes upstream of the repeats encode homologues for Cas proteins which are invariably associated with CRISPR repeats (Figure 5c). This system does share some similarities with CRISPR loci in *L. salivarius* UCC118 [48] and *Lactobacillus casei* ATCC 334 [49]. Upstream of the first *cas* gene, remnant CRISPR repeats were also identified. This phenomenon has previously been reported in *Streptococcus thermophilus* [50] and *Bifidobacterium animalis* [51] and *Bifidobacterium adolescentis* [47]. The distribution of the element in other *L. johnsonii* strains was investigated and it was indicated by PCR analysis that only the *L. johnsonii* porcine isolates DPC6092 and DPC6214 contained a similar element indicating these elements may not be widespread in *L. johnsonii* strains (Figure 4).

## Discussion

The GI tract is a complex environment that provides a variety of ecological challenges. The significant differences presented in this study highlight strain specificity among the species of the gut. Importantly based on genomic structure analysis it is suggested that the human strain of *L. johnsonii* diverged from both animal and poultry isolates at some time, however, more representative strains of each species would need to be sequenced to shed more light on this.

The chromosomal inversion, a characteristic ‘X-shaped’ symmetrical rearrangement in this study occurs within strains of the same species and based on previous analysis on closely related species it would seem that inversions across the replication axis occurs frequently in this group of Lactobacilli during evolution [15], [16]. Large genomic inversions are generally not common among bacteria of the same species but have been described in a number of pathogens such as *E. coli* [52], *Salmonella* sp. [53], *Yersinia pestis* [54], *Staphylococcus aureus* [55] and also in the non-pathogenic *Lactococcus lactis* [56]. It has been indicated that inversions may not necessarily have a selective advantage or disadvantage or dramatic phenotypic effect [56], however rearrangements have also been shown to have an effect on phenotype and cell fitness [57]. Although both strains NCC 533 (49) and DPC6026 (Figure 1) appear to have a slightly unbalanced replichore, it does not appear to have had a detrimental effect on the growth of the strains (not shown).

Despite the relative genetic homogeneity among the core regions of the sequenced *L. johnsonii* and the gene content reflecting a similar metabolic lifestyle in the GI tract, there are significant differences among adhesion proteins, mobile genetic elements and cell protection mechanisms. Notably, large differences between *L. johnsonii* isolates are in the phage complement and in putative phage resistance mechanisms. Phage integration within a replichore may influence genome stability leading to chromosomal inversions between highly conserved regions [58]. *L. johnsonii* phages Фlj965, Фlj928 [40], [41] and Фlj771 [17] have been characterised and have been shown to contribute to strain diversity within the species [17]. Фlj6026 presented in this study is integrated within the region inverted to NCC 533 and although it shares most homology with Фlj965 they are not integrated at the same site in the chromosome suggesting the phage was taken up separately by the strains and therefore it may have a particular advantage to the cell. However the functionality of this phage was not confirmed in this study. The existence of unique phage resistance mechanisms indicate that the DPC6026 genome may preferentially defend against foreign DNA integration using the CRISPR loci and/or the type III restriction modification system. As the particular elements were not found in many of the other strains tested, strain specific mechanisms for phage defence appear to be present.

It has been documented that the flora of the gut is thought to be largely modulated by the selective pressure imposed by the host and the other microbiota present [59]. As a commensal of the GI tract, *L. johnsonii* appears to be a versatile and changing bacterium that can perhaps adapts to its niche by acquiring mobile genetic elements and through chromosomal recombination events.

## Supporting Information

Figure S1 **Schematic diagram of the genetic elements at the left and right junction sites in NCC533 with reference to DPC6026.** In both junction sites, a transposase with an IS element (hatched box) and 140bp conserved sequence (filled grey box).
(TIF)

Table S1 DPC6026 specific primers used in this study.
(DOC)
