# Supplementary figures and images for: Host Specific Diversity in Lactobacillus johnsonii as Evidenced by a Major Chromosomal Inversion and Phage Resistance Mechanisms
Source: PLoS One. 2011 Apr 20;6(4):e18740. doi: 10.1371/journal.pone.0018740 (PMC3080392; doi:10.1371/journal.pone.0018740)

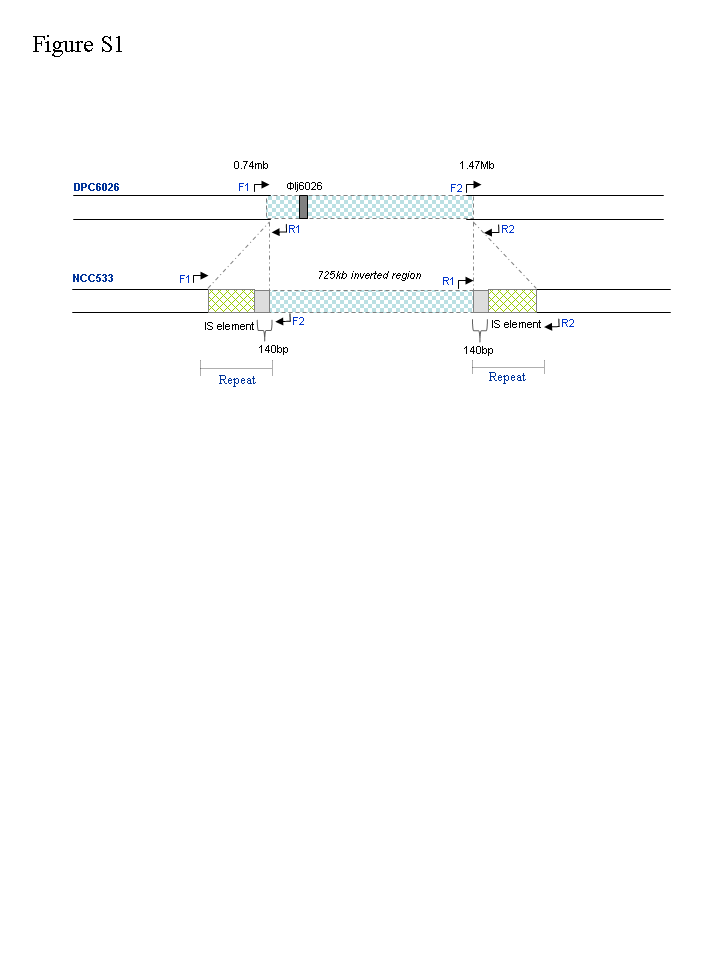

Supplement: Figure S1 — Schematic diagram of the genetic elements at the left and right junction sites in NCC533 with reference to DPC6026. In both junction sites, a transposase with an IS element (hatched box) and 140bp conserved sequence (filled grey box). (TIF) [file pone.0018740.s001.tif]
