# Supplementary material for: Host Specific Diversity in Lactobacillus johnsonii as Evidenced by a Major Chromosomal Inversion and Phage Resistance Mechanisms
Source: PLoS One. 2011 Apr 20;6(4):e18740. doi: 10.1371/journal.pone.0018740 (PMC3080392; doi:10.1371/journal.pone.0018740)
Supplement: Table S1 — DPC6026 specific primers used in this study. (DOC) [file pone.0018740.s002.doc]

**Table S1.**

| **Region** | **Primer** | **Sequence 5’-3’** |
| --- | --- | --- |
| **6026 genomic structure** | InversionF1  InversionR1  InversionF2  InversionR2 | tatattttgggggcagttgg  atgcttgcaaacaccaatga  tcttgaactccacccgaatc  tgcagaatgggttgcattta |
| **Фlj6026-specific** | IntegraseF  IntegraseR  tRNA_F  tRNA_R  Lysin F  Lysin R  LJxxxF  LJxxR | taggttttacattagtttccg  tggtggcagtgacaagagaa  cctaccttaaactgtaaagca  ctaaagttccacagtaaccac  tttatgggttgccgcttatc  gttccccacgggatatttct  tccagagccgtggctactat  gaagcgacgaggcaaattac |
| **Фlj6026-att_site** | attL_F  attL_R  attR_F  attR_R | ggccttagaaaatccgaagc  tggtggcagtgacaagagaa  cgcccgattaatttgagaaa  tttttgacaagtttatgatgcaa |
| **CRISPR_system** | Cas1_F  Cas1_R  Cas2_F  Cas2_R  Csn1_F  Csn1_R | ggtctgtgataataacacagc  tcattcggtacctcatgaattagc  gctatggtatgcagataatcgc  cagtaccaatcaacaaggtcctgat  gtactgattcatgtggatgggtag  tgcattttctgagagtattacgcc |
| **RM system** | Res_F  Res_R  Mod_F  Mod_R | ttggcttgctcaacactttg  ttgtttggcttccacaatca  gaagcaatgcagagcgtaaa  taagctgcatcacagcatcc |
